# The Variable Loop 3 in the Envelope Glycoprotein Is Critical for the Atypical Coreceptor Usage of an HIV-1 Strain

**DOI:** 10.1371/journal.pone.0098058

**Published:** 2014-06-04

**Authors:** Yue Xiang, Wei Liu, Yue Chen, Chuntao Zhang, Weiheng Su, Yan Zhang, Jiaxi Sun, Feng Gao, Chunlai Jiang

**Affiliations:** 1 National Engineering Laboratory For AIDS Vaccine, College of Life Science, Jilin University, Changchun, Jilin, China; 2 Duke Human Vaccine Institute, Duke University Medical Center, Durham, North Carolina, United States of America; 3 The 2nd Division of In Vitro Diagnostic, National Institutes for Food and Drug Control, Beijing, China; Institute of Infection and Global Health, United Kingdom

## Abstract

The majority of HIV-1 strains enter CD4^+^ T cells using the CCR5 and/or CXCR4 co-receptor. However, we recently identified a transmitted/founder (T/F) virus (ZP6248) that efficiently used an alternative coreceptor GPR15, rather than commonly used CXCR4 and CCR5, to establish clinical infection. To understand which regions in the *env* gene were critical for the atypical coreceptor usage, we generated a set of V3 mutants and determined their infectivity in GHOST cells that expressed different coreceptors. When the variable loop 3 (V3) in YU2 was replaced with the ZP6248 V3 (YU2.6248V3), the chimera YU2.6248V3 infected GPR15^+^ cells but not CCR5^+^ cells. To determine which amino acids in V3 was responsible for this phenotype change, each of the eight amino acids that differed from the subtype B consensus V3 was substituted with alanine. The G306A and S322A mutations significantly reduced the replication capacity of YU2.6248V3 in GPR15^+^ cells, while all other alanine substitutions at positions 307, 314, 315, 316, 317 and 318 completely abrogated the infectivity of YU2.6248V3 in GPR15^+^ cells. The E314A mutation, as the E314G mutation reported before, also rendered the YU2.6248V3 infectious in CCR5^+^ cells, while none of other alanine mutants could infect CCR5^+^ cells. These results demonstrated that amino acids in ZP6248 V3 might form a unique conformation that was critical for the interaction with GPR15 while the amino acids at position 314 in the V3 crown of ZP6248 played a key role in interaction with both CCR5 and GPR15. The unique phenotypes of ZP6248 can serve as a model to understand how HIV-1 explores the diverse coreceptor reservoir through novel genetic variants to establish clinical infection.

## Introduction

HIV-1 enters target cells by first binding to the primary receptor, CD4, and then one of many co-receptors. Although HIV-1 can use a number of different G protein-coupled receptors (GPCRs), the vast majority of the viruses use CCR5 and/or CXCR4 as co-receptors to infect primary cells [1]–[5]. In contrast, many simian immunodeficiency virus (SIV) strains do not use CXCR4 [6], [7], but use other co-receptors such as GPR15/BOB and Bonzo/STRL33 [7]–[9]. Moreover, frequent usage of GPR15 and STRL33 has been documented for HIV-2 [6], [10], [11]. However, studies show that HIV-1 either rarely or does not use GPR15 [12]–[15]. GPR15 is abundantly expressed on the basolateral surface of intestinal epithelium, and it mediates gp120-specific calcium signaling at low, physiologically relevant gp120 concentrations. The gp120-induced GPR15 activation was considered as a cause of HIV enteropathy [16], [17]. In addition, GPR15 regulated the homing of T cells, particularly FOXP3^+^ regulatory T cells (Tregs), to the large intestine lamina propria (LILP) [18].

Recently we identified one transmitted/founder (T/F) virus, ZP6248, which did not use CXCR4 and only used the CCR5 very inefficiently. With an unusual GPEK sequence instead of the typical GPGR crown motif in V3 of the envelope glycoprotein, ZP6248 used GPR15 very efficiently, while the V3 crown mutant E314G could enable ZP6248 to infect CCR5^+^ cells [19], suggesting that V3 plays an important role in GPR15 tropism. To further investigate which V3 domains in ZP6248 were critical for viral entry, we generated alanine substitutes for all ZP6248 V3 amino acids that are different from the subtype B consensus sequences and determined their roles in mediation of viral entry through GPR15 and CCR5.

## Materials and Methods

### Construction of the YU2 and ZP6248 V3 chimera

An overlapping PCR approach was used to generate a YU2/ZP6248 chimera by replacing the YU2 V3 with the ZP6248 V3 (YU2.6248V3). The left part genome (1507 bp) was amplified with primer-1 (5′-GACATTTTCCTAGGccatgg-3′; HXB2 nt 5653–5672), which was specific for YU2 and contained a unique *Nco*I site (small letter), and primer-2 (5′-ATATACTTTCTCTGGTCCTATATGTACACCTTTTCTTGTATTGTTGTTGGG-3′; nt 7119–7175), which was specific for the ZP6248 V3 sequence and containing all mutations that were different from subtype B consensus sequence. The right part genome (396 bp) was amplified with primer-3 (5′-GGACCAGAGAAAGTATATTTTACAACAAGCATAATAGGAGATATAAGACAAGC-3; nt 7158–7210), which was complimentary to the part of the primer-2 sequence and contained all mutations that were different from subtype B consensus sequence, and primer-4 (5′-CCCTGTAATATTTgatgaacatcta-3′; nt 7553–7577), which was specific for YU2 and contained a unique *Bsa*BI site (small letter). The complimentary regions in primer-2 and primer-3 were indicated by underline. After both fragments were independently amplified, both were purified with TIANgel Midi Purification Kit (TIANGEN, Beijing, China) and mixed together to obtain the 1858 bp fragment that contained the YU2 *env* gene with the ZP6248 V3 sequence using primer-1 and primer-4. The PCR was carried out with Phusion Hot Start DNA polymerase (Finnzymes, Espoo, Finland) to minimize the misincoporations during PCR. The following thermal cycling conditions were used: denaturation at 98°C for 30 sec, followed by 30 cycles of 98°C for 15 sec, 50°C for 30 sec, and 72°C for 1 min. The resulting PCR fragment was purified, digested and cloned into YU2 at the *Nco*I and *Bsa*BI sites to generate YU2.6248V3. The final clone was confirmed by sequencing.

### Site-directed mutagenesis

The partial *env* gene containing ZP6248 V3 was amplified from YU2.6248V3 using primer-1 and primer-4 and cloned into the pGEM-T easy vector (Promega, Madison, WI, USA). To replace the codons for the ZP6248 V3 amino acids that differed from the subtype B consensus sequence with the Ala codon, site-directed mutagenesis was carried out using the Quikchange Site-directed Mutagenesis kit (Stratagene, La Jolla, CA, USA). Briefly, each mutagenesis reaction contained 1x reaction buffer, 1 µl dNTP mix, 5–50 ng of plasmid DNA, 10 µM of each primer, 1 µl PfuTurbo DNA Polymerase, and double distilled water to a final volume of 50 µl. The mutagenesis reaction was performed under the following conditions: denaturation at 95°C for 30 sec; 18 cycles of 95°C for 30 sec, 55°C for 1 min; and 68°C for 4 min 55 sec. The *Dpn*I endonuclease (Takara, Otsu, Shiga, Japan) was then used to digest the parent template DNA strands. Five microliters of the *Dpn*I-digested reaction solution was used to transform JM109. The *Nco*I and *Bsa*BI fragments that contained the Ala substitutions were cloned into the YU2 clone at the *Nco*I and *Bsa*BI sites. Eight YU2.6248V3 Ala mutants were generated (G306A, V307A, E314A, K315A, V316A, Y317A, F318A and S322A).

The unique ZP6248 GPEK V3 crown was introduced into the YU2 (YU2.GPEK) by site-directed mutagenesis. In addition, amino acids at positions 11 and 25 in V3 that play a critical role in determination of the CCR5 or CXCR4 were also mutated, individually or in combination (YU2.GPEK-G306, YU2.GPEK-S322 and YU2.GPEK-G306/S322). The amino acid at position 24 in V3 was deleted in ZP6248. Thus, both the deletion at position 24 and a mutation at position 25 (E322S) were also introduced. To more efficiently introduce the mutations into YU2.GPEK, the YU2 V3 fragment was first cloned into the pGEM-T-easy vector (pGEM-T-YU2.V3). After site-directed mutagenesis was confirmed by sequencing, the mutated V3 fragments were cloned back into the YU2 clone at the *Nco*I and *Bsa*BI sites. All final mutant clones were confirmed by sequencing.

### Virus stocks

HEK293T cells (ATCC, Manassas, VA, USA) were cultured in Dulbecco's Modified Eagle's Medium supplemented with 10% fetal bovine serum at 37°C. The plasmid DNA (15 µg) was transfected into HEK293T cells in a T75 flask using lipofectamine 2000 (Invitrogen, Carlesbad, CA, USA). Forty-eight hours after transfection, the cell culture supernatants were harvested and stored at −80°C for future use.

### Western blotting analysis

The cell culture supernatants from transfected HEK293T cells were pelleted through a 20% sucrose cushion at 130000 g for 2 h at 4°C. The pelleted virus particles and transfected HEK293T cells were lysed separately with 300 µl of the lysing buffer (50 mM Tris-HCl, 150 mM NaCl, 1% Triton X-100, 0.1% SDS, pH 7.4) and 100 µl 4×protein SDS-PAGE loading buffer (40 mM Tris-HCl, 200 mM DTT, 4% SDS, 40% Glycerol, 0.032% Bromophenol Blue). The virus and cell lysates were heated at 97°C for 10 min and then loaded onto a 10% SDS-PAGE gel. The separated proteins were then transferred to a nitrocellulose membrane and blocked in PBS containing 3% dry skim milk for 1 h. The blotted proteins were reacted with purified immunoglobulin from HIV-1^+^ sera (HIVIG; Quality Biological, Gaithersburg, MD, USA). Finally, the membranes were reacted with IRDye 800CW goat anti-human IgG (H+L) and images were acquire using Odyssey (LI-COR, Lincoln, NE, USA).

### Determination of co-receptor usage

GHOST cells (NIH AIDS Reagent Program, Division of AIDS, National Institute of Allergy and Infectious Diseases, National Institutes of Health) were seeded at 2×10^5^ per well in 12-well plates the day before infection. Equal amount of each virus (5 ng p24) was added to each well. After absorption for 6 h at 37°C, the cells were washed three times and resupplemented with complete medium. The cell culture was maintained for 10 days. Cell culture supernatants were collected every two days to monitor viral replication kinetics by determining the p24 concentrations using the Alliance HIV-1 p24 Antigen ELISA kit (PerkinElmer, Waltham, MA, USA).

### Cell viability assay

Cellular ATP was measured in white 96-well plates using the CellTiter Glo luminescence ATP assay kit (Promega, Madison, WI, USA). One hundred microliters of GHOST cells (10^4^ cells per well) were seeded in 3 replicate wells and infected with 0.5 ng of each virus per well. After absorption for 6 h at 37°C, the cells were washed and resupplemented with complete medium. The cell culture was maintained for 10 days when the cells were lysed and the ATP concentration was detected as relative light units (RLU) using a luminometer (PerkinElmer 2030 Multilabel Reader, Waltham, MA, USA).

### Statistical analyses

The susceptibilities to virus infection and cell viabilities of GHOST cells were compared using the student *t* test. All statistical analyses were performed using SPSS version 21.

## Results

### The GPR15 tropism was determined by V3 in ZP6248

**Figure 1:**
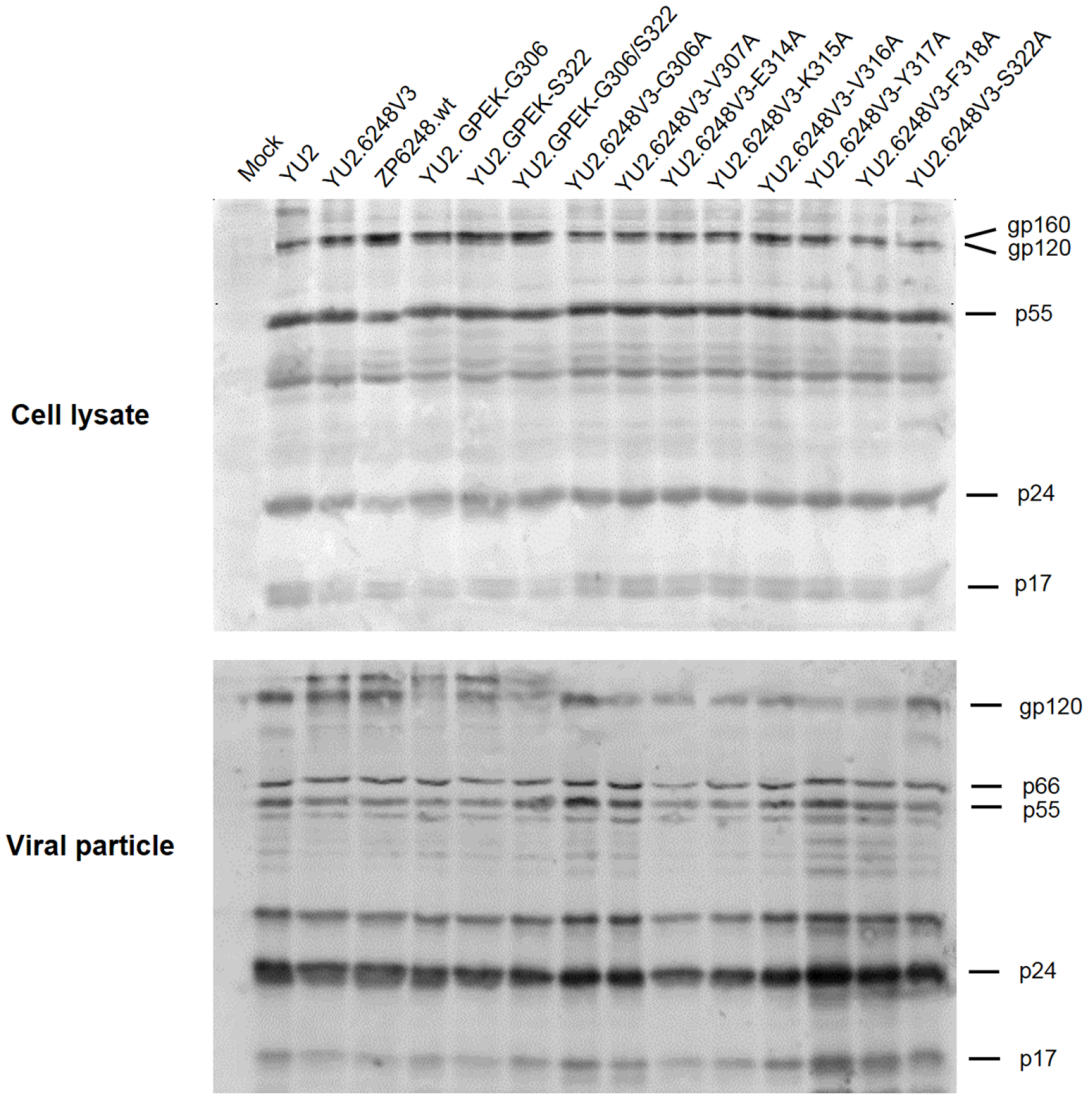
Analysis of viral proteins in transfected cells and viral particles. HEK293T cells were transfected with plasmid DNA. Two days after transfection, the transfected cells and supernatants were harvested and lysed in lysing buffer as previous described [19]. The lysates were then separated on a 10% SDS-PAGE gel and viral proteins were examined by Western blotting using the purified immunoglobulin from HIV-1^+^ sera.

**Figure 2:**
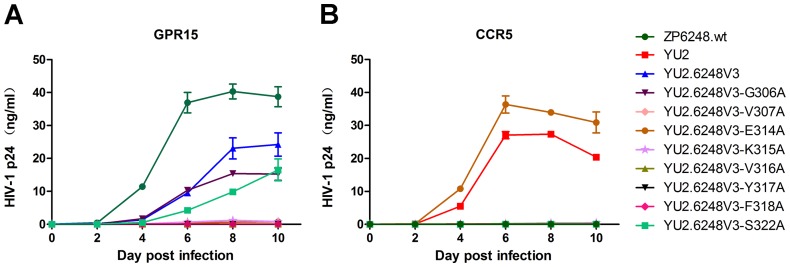
Replication kinetics of the ZP6248 V3 chimera and mutants. GHOST GPR15 (A) and CCR5 (B) cells were infected with the same amount (5 ng p24) of each virus and the viral replication was monitored by measuring the p24 concentrations in the culture supernatants. Each virus was assayed in triplicate and mean ± standard deviation is shown. Similar results were obtained in two independent experiments and the results from one experiment are shown.

We have previously shown that GPR15 tropism of ZP6248 was retained when its *env* gene was substituted into a CCR5-tropic HIV-1 strain YU2 [19]. To determine if the V3 from ZP6248 alone could confer GPR15 tropism, we substituted the YU2 V3 with the ZP6248 V3 (YU2.6248V3). The substitution of the ZP6248 V3 did not affect the expression of Env in the cell and the incorporation of gp120 into the virus particles (Fig. 1). YU2.6248V3 replicated well in GPR15^+^ cells although at a relatively low level compared to ZP6248.wt (Fig. 2A). The ZP6248 V3 also rendered YU2 not infectious in CCR5^+^ cells (Fig. 2B). These results were similar to that observed with the ZP6248 Env pseudotyped virus [19]. Thus, the use of GPR15 and the inefficient use of CCR5 by ZP6248 were determined by V3.

### The unique amino acids in the ZP6248 V3 play a critical role in interaction with GPR15

**Figure 3:**
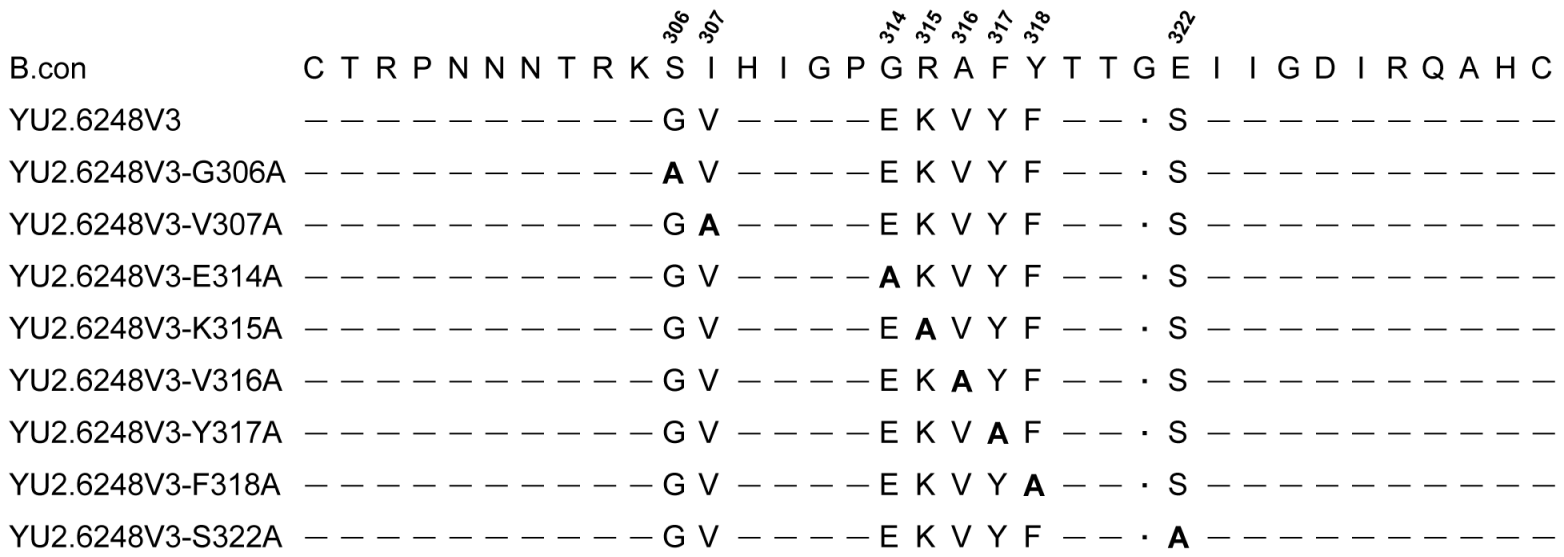
Sequence alignment of the ZP6248 V3 chimera and mutants. The ZP6248 V3 sequence was aligned to the HIV-1 subtype B consensus sequence (B.con). Alanine substitutions (bold) were made at the positions where amino acids are different between ZP6248 and the B.con sequence. The amino acids identical to the B.con sequence are shown as dashes and the dot indicates the deletion.

To investigate which of the unique amino acids in V3 was critical for ZP6248 to use GPR15 for entry, we individually replaced all eight amino acids that were different from the subtype B consensus sequence with alanine (Fig. 3). None of the alanine substitution mutants affected the expression of Env in the cell and the incorporation of gp120 into the viral particles (Fig. 1). Six alanine mutants (V307A, E314A, K315A, V316A, Y317A and F318A) completely lost their ability to infect GPR15^+^ cells (Fig. 2A), suggesting that they were critical for interacting with GPR15. The p24 concentrations in the culture of two alanine mutants (G306A and S322A) were similar but slightly lower than that of YU2.6248V3 in GPR15^+^ cells (Fig. 2A).

**Figure 4:**
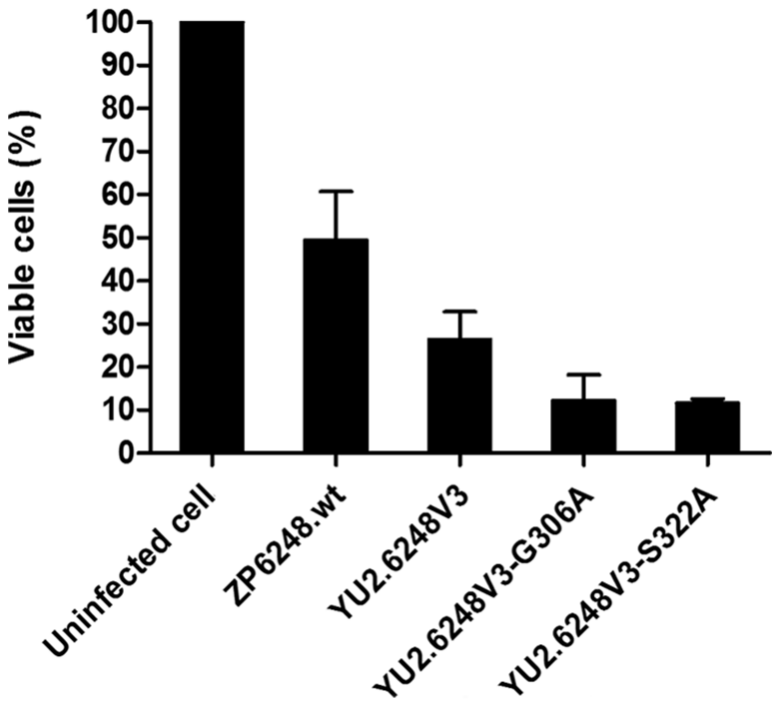
Viability of cells infected with ZP6248 and its mutants. The GHOST GPR15 cells were infected with ZP6248 and its mutants and cultured for 10(day 10) was determined by CellTiter-Glo Luminescent Cell Viability Assay. Each virus was assayed in triplicate and mean ± standard deviation is shown. Similar results were obtained in two independent experiments and the results from one experiment are shown.

Examination of the cell viability showed that wild type ZP6248 was cytopathic in GPR15^+^ cells. The cell death was first observed at day 4 in the culture (Table 1) and about 50% of the cells died at day 10 (Fig. 4). The ZP6248 V3 rendered YU2 more cytopathic than the wild type ZP6248 (p = 0.001); only 26% of cells were still viable in the YU2.ZP6248V3 culture at day 10 (Fig. 4). Both G306A and S322A mutants caused significantly more cell death than wild type ZP6248 (p<0.001) as well as YU2.6248V3 (p = 0.003). The cell death was observed at day 2 in the culture (Table 1) and about 90% of the cells died at day 10 (Fig. 4). These results suggested that the low levels of p24 detected in the culture of YU2.6248V3 as well as both G306A and S322A mutants in GPR15^+^ cells was partly due to the higher numbers of dead cells.

**Table 1:** Cytopathic effect of ZP6248 and its mutants in GHOST GPR15 cells.

Virus	Day 2	Day 4	Day 6	Day 8	Day 10
ZP6248.wt	−	+	+	++	++
YU2.6248V3	−	+	++	+++	+++
YU2.6248V3-G306A	+	++	+++	++++	++++
YU2.6248V3-S322A	+	++	+++	++++	++++
No virus	−	−	−	−	−

% of dead cells: – (0%); + (1%–25%); ++ (26%–50%), +++ (51%–75%) and ++++ (76%–100%).

Among all eight alanine mutants, only E314A mutant could render the YU2.6248V3 chimera to infect CCR5^+^ cells (Fig. 2B). In our previous study, the E314G mutation also allowed ZP6248 to efficiently use CCR5 for entry [19]. Taken together, these results demonstrated that the majority of unique amino acids in the ZP6248 V3 crown might form a conformation that was critical for the interaction with GPR15 while the amino acid at position 314, in the context of the ZP6248 V3 sequence, played a critical role in efficient interaction with CCR5 and GPR15.

### Mutations at positions 11 and 25 in V3 could not render the YU2.GPEK mutant infectious in GPR15^+^ cells

**Figure 5:**
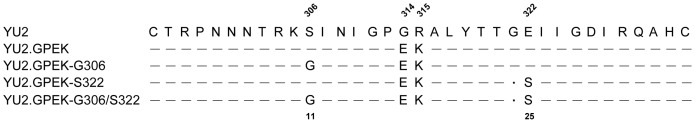
Sequence alignment of the YU2 V3 mutants. The YU2 V3 mutant sequence was compared to the wild type YU2 sequence (top line). The positions 11 and 25 in V3 (below the alignment) that correspond the position 306 and 322 in gp160 (above the alignment) are indicated. The identical amino acids to the YU2 sequence are indicated by dashes and the dots indicate the deletion.

**Figure 6:**
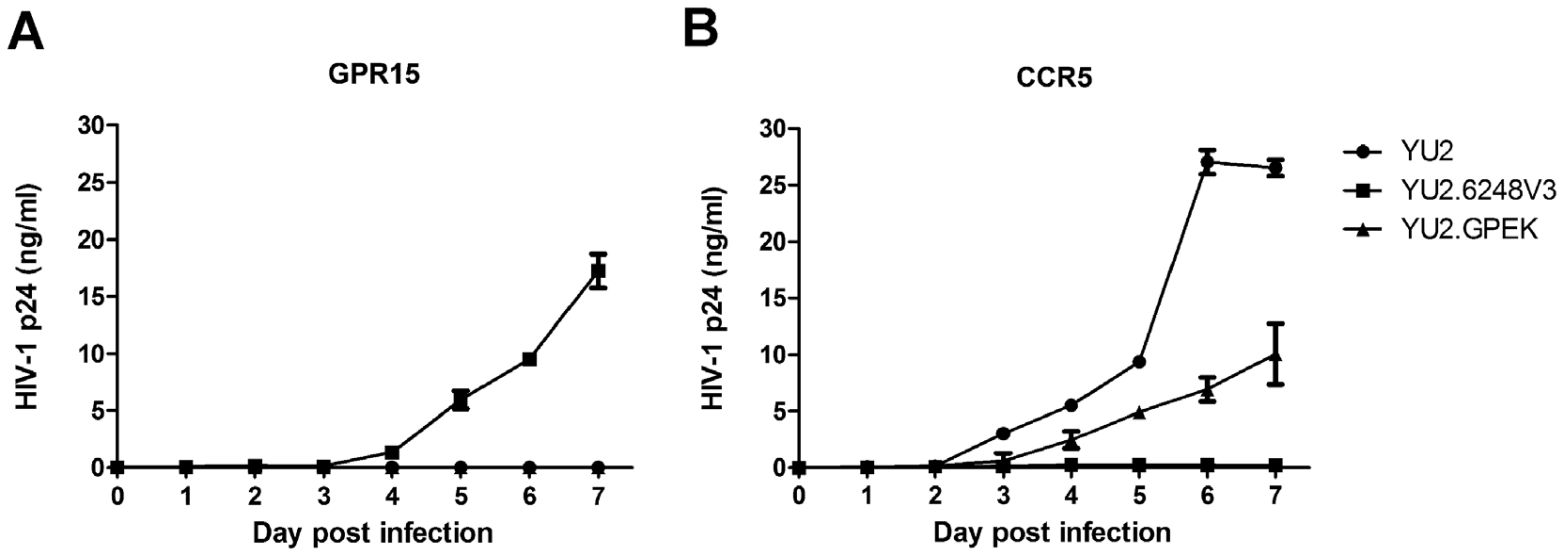
Replication kinetics of YU2 mutants containing amino acids unique for ZP6248. GHOST GPR15 (A) and CCR5 (B) cells were infected with the same amount (5 ng p24) of each virus and the viral replication was monitored by measuring the p24 concentrations in the culture supernatants. Each virus was assayed in triplicate and mean ± standard deviation is shown. Similar results were obtained in two independent experiments and the results from one experiment are shown.

Since both the E314G and E314A mutation could reduce the replication capacity of ZP6248 in GPR15^+^ cells but allowed the virus to infect CCR5^+^ cells, we sought to test if introduction of the rare combination of glutamic acid and lysine into the YU2 (YU2.GPEK) would allow virus to infect GPR15^+^ cells (Fig. 5). YU2.GPEK did not replicate in GPR15^+^ cells (Fig. 6A) but its replication capacity was significantly impaired in CCR5^+^ cells (Fig. 6B), suggesting that the unique GPEK V3 crown motif alone could not allow a heterologous virus to infect target cells using GPR15. Both G306A and S322A mutants only partially impaired ZP6248′s ability to infect GPR15^+^ cells (Fig. 2A). Those two mutations were also at positions 11 and 25 in V3 (Fig. 5), which are known to be important to modulate the usage of CCR5 and CXCR4 coreceptors [20], [21]. To test if the replacement of those mutations in YU2.GPEK with those in ZP6248 would render YU2.GPEK infectious in GPR15^+^ cells, we generated three additional mutants by introducing, individually or in combination, glycine and serine that were present in ZP6248 at positions 11 and 25 into YU2.GPEK (Fig. 5). Both individual mutants (YU2.GPEK-G306 and YU2.GPEK-S322) as well as the double mutant (YU2.GPEK-G306/S322) were not able to infect GPR15^+^ cells and they all abrogated the YU2.GPEK's ability to replicate in CCR5^+^ cells. These results demonstrated that the combination of unique amino acids at positions 306, 314 and 322 in ZP6248 were not sufficient to allow heterologous viruses to infect target cells through GPR15.

## Discussion

In contrast to all other HIV-1 T/F viruses that use either CCR5 and/or CXCR4 coreceptors for entry, the T/F virus ZP6248 established the clinical infection most likely used the coreceptor GPR15 and the unique V3 crown GPEK in ZP6248 play a critical role in the GPR15 tropism [19]. By generating a serial of mutants, we now more precisely defined the regions that were critical for ZP6248 to infect GPR15^+^ cells. The ZP6248 V3 alone could effectively mediate viral entry through GPR15, but the replication capacity of YU2.6248V3 was lower than that of the wild type ZP6248. This suggested that the ZP6248 tropism for GPR15 was mainly determined by the V3 although sequences outside V3 might be also required for optimal infection of GPR15^+^ cells. This is similar to the observations that regions outside V3 also play a role in determine the optimal use of CCR5 and/or CXCR4 [22]–[25].

Mutations at six of eight sites that differed from those in subtype B consensus sequence could completely render the YU2.6248V3 chimera non-infectious in GPR15^+^ cells. Analysis of 6,010 Env sequences revealed highly conserved three-dimensional structures of V3 [26], including residues at the base of the β-hairpin (Zone 1), the hydrophilic domain (Zone 2), the hydrophobic domain (Zone 3) and the turn of the peptide chain (Zone 4). All but one of these eight sites were in the hydrophobic domain Zone 3 (^305^KRKRIHIGPGRAFYTT^320^). Thus, alanine substitutions of these amino acids could have altered the conformation of V3 and made the mutants unable to enter the target cells through GPR15. Introduction of the unique GPEK V3 crown sequence into a R5 virus YU2 did not enable the mutant YU2.GPEK to use GPR15. Inclusion of two additional mutations (G306 and/or S322), which were at the same positions as 11 and 25 in V3 and played a critical role in modulation of the CCR5 and CXCR4 usage, completely abrogated YU2.GPEK's ability to replicate in CCR5^+^ and GPR15^+^ cell. This result indicated that the amino acids unique for ZP6248 each alone were not sufficient to render a heterologous virus to gain the ability to use GPR15. However, all those unique amino acids together in ZP6248 V3 were critical in mediating infection thorough the use of GPR15. Taken together, our results suggested that unique amino acids in ZP6248 V3 together might form a conformation that favored the use of GPR15, but not CCR5 or CXCR4, for viral entry.

In our previous study, we have found that the single E314G mutation in the V3 crown tip of ZP6248 could partially restore its infectivity in CCR5^+^ cells, but reduced its ability to replicate in GPR15^+^ cells [19]. The E314A mutant had similar phenotypes, but infected CCR5^+^ cells at a higher level. The E314A mutant replicated in CCR5^+^ cells slightly better than YU2 but failed to grow in GPR15^+^ cells. These results demonstrated that the different amino acids at position 314 played an important role in modulating usage of different coreceptors. Other studies showed that some viruses could infect target cells using GPR15 [7], [13], [27], [28]. However, unlike ZP6248, all those viruses mainly used CCR5 and/or CXCR4 for entry. Examination of the V3 sequences of those viruses showed that they had the typical GPGR V3 crown motif. The lack of the unique GPEK V3 crown motif in those viruses might explain why they infected the target cells mainly through the commonly used CCR5 and/or CXCR4 instead of GPR15 like ZP6248.

The cytopathic effect of the YU2/ZP6248 V3 chimera (YU2.6248V3) was significantly stronger than the wild type ZP6248 in the GHOST GPR15 cells. More severe cell death was observed in cells infected with either YU2.6248V3-G306A or YU2.6248V3-S322A mutants. These results suggest that a more pathogenic virus might be made when a recombinant virus between ZP6248 and a heterologous virus was generated. Additional studies are needed to understand the mechanisms for the cell death caused by these mutants.

In conclusion, the atypical coreceptor usage of ZP6248 was mainly determined by the majority of the unique amino acids in V3 of the envelope glycoprotein. Since SIVrcm from red-capped mangabeys often uses CCR2 as a coreceptor due to the absence of CCR5 [29]–[31] and since SIVsmm from sooty mangabeys can also use a coreceptor other than CCR5 to mediate infection *in vivo* [32], [33], it is possible that the unique variants like ZP6248 may become prevalent in humans, especially in the face of use of CCR5 antagonists, which may lead to the selection and spread of viruses that are able to establish infection without using CCR5. More alarmingly, the ZP6248 V3 in YU2 was significantly more cytopathic. If such recombinants occur *in vivo*, a more virulent variant may be generated. Therefore, it is important to investigate the novel infection mechanisms required for the virus to establish clinical infections and continue to monitor ZP6248-like viruses in humans. Moreover, the unique phenotypes of ZP6248 can serve as a model to understand how HIV-1 explores the diverse coreceptor repertoire through novel genetic variants to establish clinical infection.
